# Characterization of rhesus macaque model for cobalt-60 gamma-radiation source without use of blood product

**DOI:** 10.1038/s41598-025-17099-7

**Published:** 2025-08-26

**Authors:** Stephen Y. Wise, Oluseyi O. Fatanmi, Sarah A. Petrus,, Issa Melendez-Miranda, Matthew Brink, Benjamin Packer, Alana D. Carpenter, Thomas M. Seed, Vijay K. Singh

**Affiliations:** 1 Division of Radioprotectants, Department of Pharmacology and Molecular Therapeutics, F. Edward Hébert School of Medicine, Uniformed Services University of the Health Sciences, 4301 Jones Bridge Road, Bethesda, MD 20814 USA; 2 Armed Forces Radiobiology Research Institute, Uniformed Services University of the Health Sciences, Bethesda, MD 20814 USA; 3 Tech Micro Services, 4417 Maple Avenue, Bethesda, MD 20814 USA

**Keywords:** Gamma-radiation, Rhesus macaque, Survival, Total-body irradiation, Therapeutics, Drug discovery

## Abstract

Despite significant radiobiological advancements following World War II, only a limited number of medical countermeasures (MCMs) have been approved by the United States Food and Drug Administration (US FDA) for acute radiation exposure related illnesses. Accordingly, well-characterized and validated animal models, both large and small, are still very much needed to develop safe and effective countermeasures. Animal models that are used for such purposes need to reflect not only the clinical and pathogenic features of those seen in radiation exposed humans, but also comparable radiation dose- and time-dependent relationships. The objective of the present study therefore was to further characterize the response patterns of rhesus nonhuman primates exposed to total-body, potentially lethal, radiation doses using the Armed Forces Radiobiology Research Institute high level cobalt-60 gamma-radiation source. Response patterns of male and female rhesus macaques were assessed following acute, total-body exposures to potentially lethal, gamma rays (5.8, 6.5, and 7.2 Gy). Groups of 15, 16, and 8 animals were exposed to the three radiation doses, respectively. All animals were provided a minimum, subject-based supportive care, that excluded the use of blood products. Blood products were excluded in order to replicate a large scale radiological/nuclear scenario treatment option in which access to blood products may be limited or unavailable. This is also relevant for military scenarios, in which medical facilities may not have the appropriate capabilities for blood transfusions. All animals were clinically monitored for 60 days post-irradiation. Survival was the primary endpoint of this study, while secondary endpoints included recovery of various hematopoietic elements. The mortality rates of the rhesus macaques were 33%, 37.5% and 50%, respectively, for the three radiation doses (i.e., 5.8, 6.5 and 7.2 Gy). Within the surviving animals, hematological blood values had returned largely to pre-exposure levels by the end of the study period. The results of this study provides foundational data on the use of the rhesus macaque model for subsequent development and testing of new radiation MCMs, as per required by the US FDA Animal Rule.

## Introduction

Over the last several decades, there has been unequivocal increases in both terrorist activity, and the world-wide dissemination of nuclear materials^1^. Consequentially, the potential risk associated with unwanted radiation exposures has substantially increased for both civilians and military personnel alike. Such radiation exposures may result in the development of a complex of radiation exposure-related diseases that often result in substantial morbidity and mortality^2^. High-dose radiation exposure over a short period of time can cause various types of injuries, necessitating diagnostic and therapeutic intervention to save lives. One such prominent indication, the so called ‘acute radiation syndrome’ (ARS) and its progression following exposure depends on several radiobiological factors, including the absorbed dose, dose rate, radiation quality, and the distribution of radiation within the body. In humans, ARS typically develops after total-body or partial-body exposure at doses generally exceeding 1 Gy, especially when delivered at relatively high dose rates (> 0.5 Gy/h). Clinically, several sub-syndromes of ARS may present as a result of varying conditions of exposure^3^. Neurovascular ARS, induced by supralethal doses of radiation, is generally considered untreatable, with death potentially occurring within 24 – 48 h. However, individuals exposed to radiation doses between 1 to 10 Gy are more likely to respond favorably to the treatment options of radiation medical countermeasures (MCMs). Currently, all of the United States Food and Drug Administration (US FDA)-approved MCMs for ARS are specifically for H-ARS and these agents are used as post-exposure radiomitigators. These include Neupogen, Neulasta, Leukine, and Nplate, along with their seven biosimilars: four for Neulasta (Udenyca, Stimufend, Fylnetra and Ziextenzo) and three for Neupogen (Nypozi, Releuko and Zarxio)^4–15^. No agent has been approved for GI-ARS, either as a prophylactic drug or as a post-exposure treatment.

The large animal model of nonhuman primates (NHPs) is generally considered as a ‘gold standard’ for regulatory approval of drugs by the US FDA due to their close resemblance to human anatomy, genomic activity, pathways, and biological targets. The most frequently used NHPs in biomedical/radiobiological-related research investigations are rhesus macaques^16^. However, due to market demand, the availability of these animals for radiobiological research has become quite limited and hence problematic. Rhesus macaques have been extensively used to define ionizing radiation injuries caused by a wide variety of exposure conditions (dose, dose-rate, radiation quality, extent of bodily exposure, etc.), as well as to evaluate the efficacy of MCMs for regulatory approval^5,6,9,14,17,18^. This is because rhesus macaques possess several characteristics that make them more akin to humans than the vast majority of other laboratory animal species. These favorable traits most certainly include, but are not limited to: extended life spans, anatomical and physiological similarities of various organ systems to that of humans, and the equivalences of basic pathologic responses of various organ systems to ionizing radiation. Furthermore, rhesus macaques not only share close genetic features to humans, as indicated by its ~ 95% DNA sequence homologies, but have similar metabolisms. Due to their relatively large size, sequential sample collection during the course of an extended study is fairly simple and straight forward. These close associations to humans have made rhesus NHPs appealing animal models for the development and approval of MCMs following the US FDA Animal Rule.

A well-characterized animal model that includes the possible use of various types of exposure conditions is absolutely essential for MCM development, as well as for identifying and validating biomarkers of radiation damage and advancing biodosimetry^19,20^. This strategy is clearly supported by earlier studies that stem from various institutions that have used different radiation sources, experimental conditions, exposure types, levels of supportive care, and various qualities of radiation for the experimental induction of H-ARS in various animal models, but most specifically in rhesus macaques^21–23^. However, there have been only a limited number of studies reported (e.g., using the Armed Forces Radiobiology Research Institute (AFRRI)) that have used total-body, ^60^Co gamma ray exposures to induce H-ARS in rhesus macaques and to study lethality patterns within those animals without the use of supplemental, supportive care in the form of transfused blood products. Such studies are deemed important as they represent critical baseline studies relative to establishing the radiation dose-dependencies of the lethality responses of these rhesus macaques and for their use in determining MCM efficacies. This experimental situation closely resembles the austere medical management likely to be available after a mass casualty nuclear detonation versus a small-scale radiation accident. This also has relevance to emergency management on the front line within DoD or in a civilian setting.

We conducted here a study that serves to further characterize the lethality and hematological response patterns of male and female rhesus macaques under three different, near lethal doses of total-body γ-radiation using a relatively high rate of exposure (0.6 Gy/min), and with limited supportive care provided (i.e., no transfused blood products were used). Mortality and moribundity were assessed over a 60-day post-exposure period and served as primary endpoints. These characterizations of the rhesus NHP model are crucial for determining the efficacy of radiation MCMs for regulatory approval by the US FDA under the Animal Rule^21,24,25^.

## Results

### Change in vital clinical parameters following potentially lethal levels of radiation exposure

**Fig. 1:**
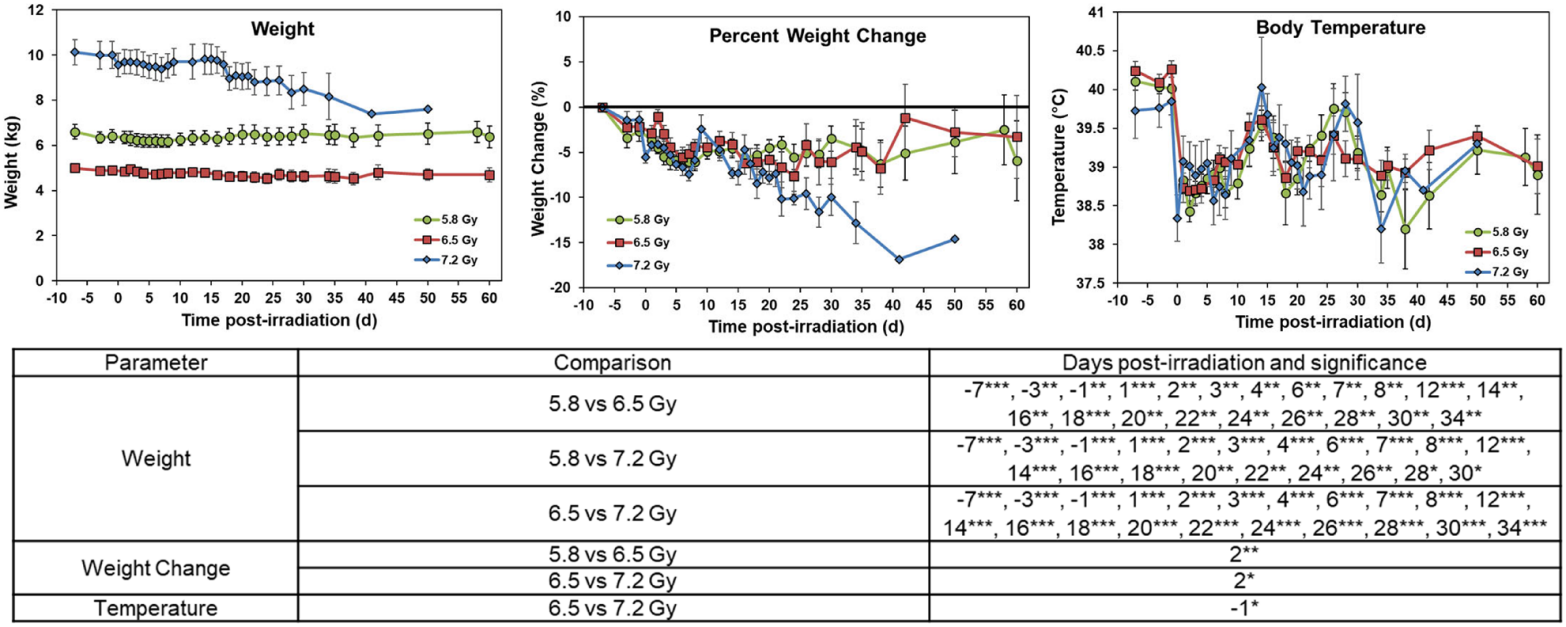
Effects of 5.8 (n = 15), 6.5 (n = 16), or 7.2 (n = 8) Gy ^60^Co total-body γ-radiation on body-weight, body-weight percent change, and body temperature. Data for each time point are presented as the mean for each group. Statistical significance is presented in the table below the graphs and are denoted by asterisks where *p < 0.05, **p < 0.01, and ***p < 0.001. Error bars represent SEM.

Animal weight, weight percent change, and body temperature were measured throughout the study, which are presented in Fig. 1. Significant differences in weight were observed between all dose groups at most timepoints. However, it is important to note that the average weights of the animals in each radiation dose group had distinctly different pre-irradiation weights. This is due to these experiments being conducted sequentially, and not concurrently. The animals were procured at different times and therefore had different average starting body weights. The body-weight percent change within each group compared to pre-irradiation timepoint has also been shown (upper middle panel of Fig. 1). Body temperature showed only a few significant differences between the three dose groups.

The 5.8 and 6.5 Gy exposure groups displayed similar patterns in body weight and body weight percent change, with marginal decreases after irradiation that returned to baseline levels by day 60. The 7.2 Gy exposure group seemed to be more affected by radiation in these parameters, and had a much sharper decrease in weight, with weight not returning to baseline levels by the end of the study. Body temperature remained similar between all radiation dose groups, with no consistent patterns observed following irradiation (Fig. 1).

### Effects of potentially-lethal radiation doses on mortality rates

**Fig. 2:**
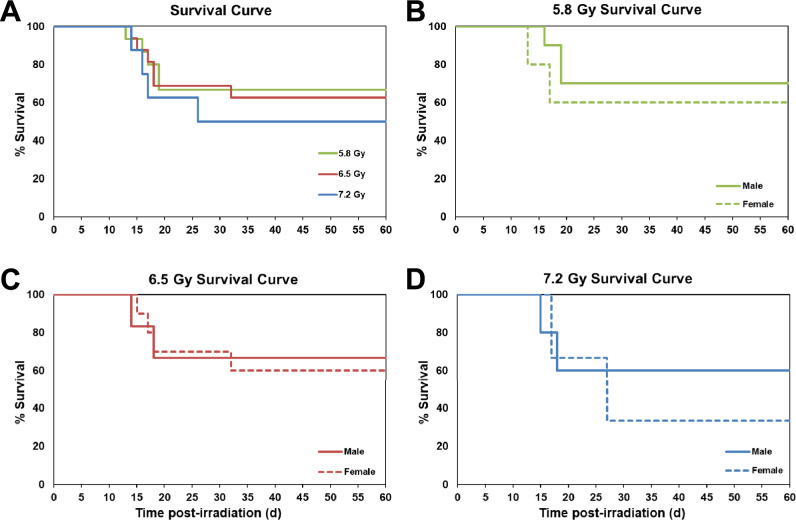
Kaplan–Meier curves of irradiated NHPs. Thirty-nine NHPs were exposed to either 5.8 (n = 15), 6.5 (n = 16), or 7.2 (n = 8) Gy ^60^Co total-body γ-radiation at a dose rate of 0.6 Gy/min. Survival rates were monitored for 60 days post-irradiation. Panel (**A**): Combined survival curve of NHPs exposed to 5.8 Gy, 6.5 Gy, or 7.2 Gy. Panel (**B**): Survival curve of 5.8 Gy exposed male (n = 10) and female (n = 5) NHPs. Panel C: Survival curve of 6.5 Gy exposed male (n = 6) and female (n = 10) NHPs. Panel D: Survival curve of 7.2 Gy exposed male (n = 5) and female (n = 3) NHPs.

Rates of mortality at the end of the defined test period (60 days post-irradiation) marginally increased, but not statistically significantly, as the extent of exposure (i.e., the radiation dose) increased from 5.8 Gy to 6.5 Gy to 7.2 Gy (Fig. 2, Panel A). Specifically, the 5.8 Gy exposed group of animals exhibited the lowest mortality rate of 33% (or conversely the highest survival rate of 67%), whereas the animals exposed to the higher doses of 6.5 and 7.2 Gy had slightly, but progressively increased mortality rates of 37.7% and 50%, respectively (Fig. 2, Panel A). The majority of animals died between d 12 and 20 post-irradiation. Separate data analysis for decedents and the comparison of such animals with 60-d survivors is planned for a publication in the future. Though this study is considered mixed sex, the ratio of males and females in each group was not the same. Survival curves showing male and female mortality following exposure to 5.8, 6.5, and 7.2 Gy are presented as Fig. 2, Panels B, C, and D, respectively. Females in all three dose groups had higher mortality, however, these results were not found to be statistically significant, potentially due to the small sample size.

### Effects of potentially lethal radiation exposures on blood cell counts over the experimental time course

**Fig. 3:**
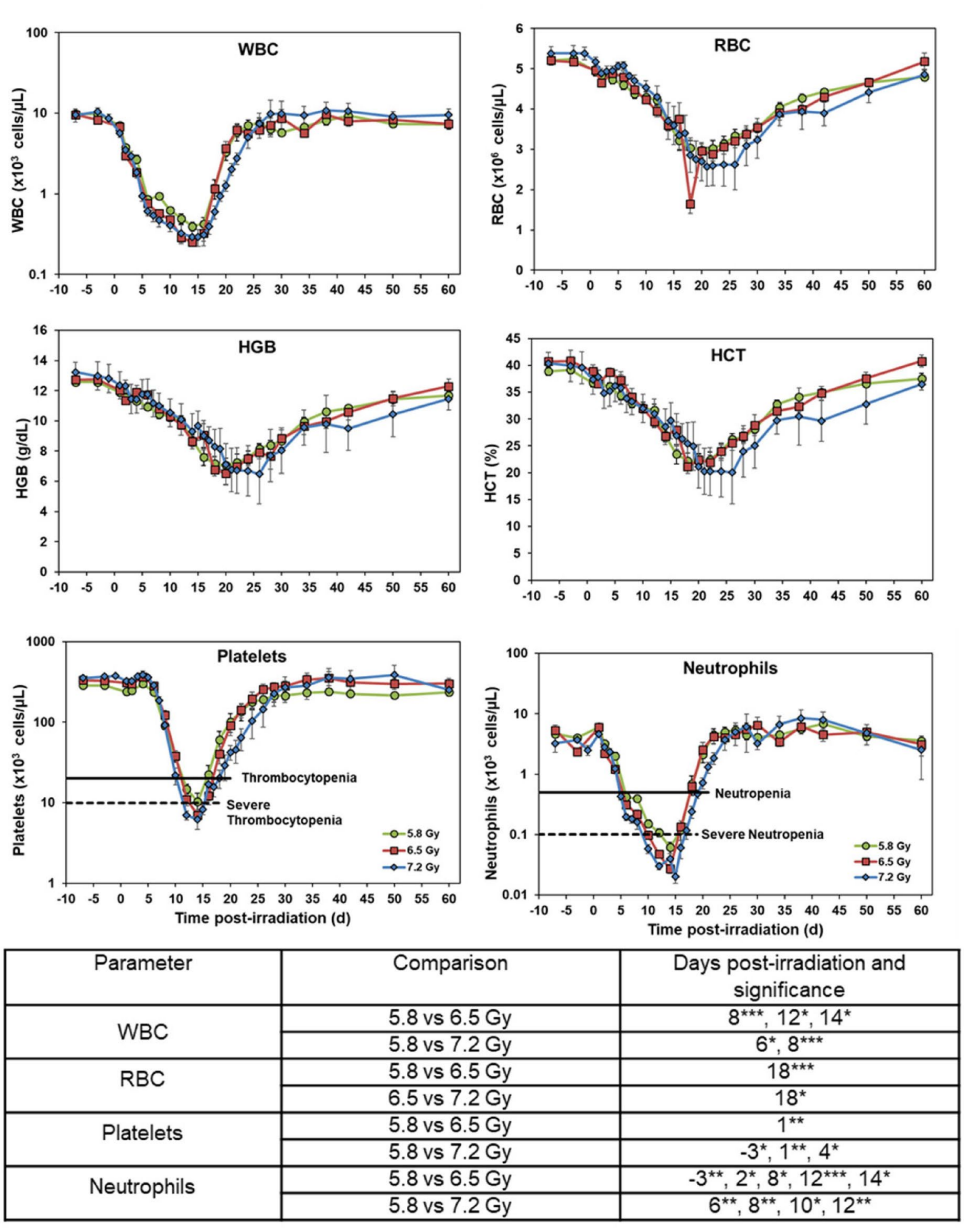
Effects of 5.8 (n = 15), 6.5 (n = 16), or 7.2 (n = 8) Gy ^60^Co total-body γ-radiation on white blood cells (WBC), red blood cells (RBC), hemoglobin (HGB), hematocrit (HCT), platelets, and neutrophils. Data for each time point are presented as the mean for each group. Statistical significance is indicated in the table below the graphs, denoted by asterisks, where *p < 0.05, **p < 0.01, ***p < 0.001. Error bars represent SEM.

**Fig. 4:**
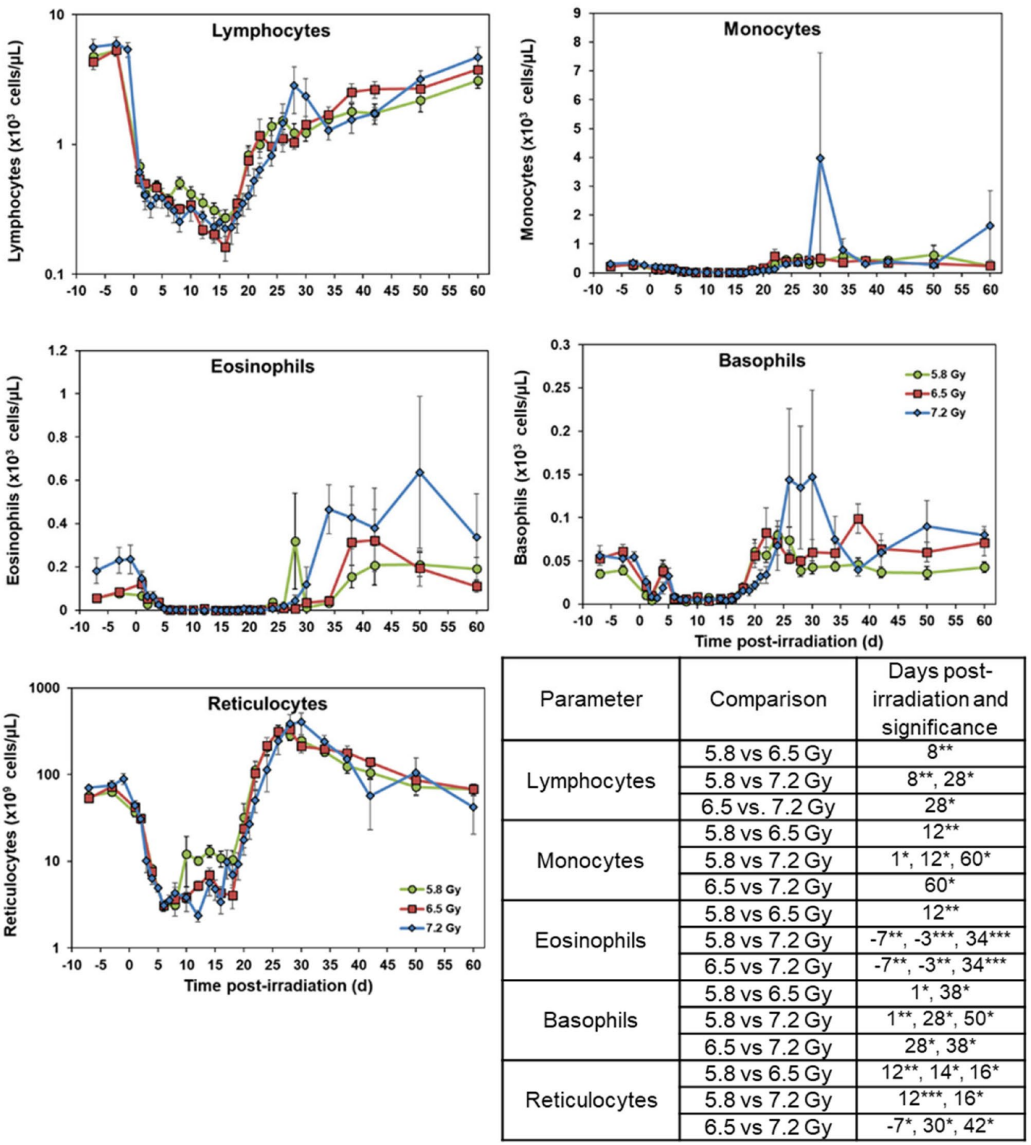
Effects of 5.8 (n = 15), 6.5 (n = 16), or 7.2 (n = 8) Gy ^60^Co total-body γ-radiation on lymphocytes, monocytes, eosinophils, basophils, and reticulocytes. Data for each time point are presented as the mean for each group. Statistical significance is indicated in the table below the graphs and are denoted by asterisks where *p < 0.05, **p < 0.01, and ***p < 0.001. Error bars represent SEM.

Eleven parameters were analyzed at various time points pre- and post-irradiation, which are shown in Figs. 3 and 4 (X-axis denotes the days). Significant differences were observed between the radiation dose groups for most parameters, with all parameters returning to baseline or near-baseline levels by day 60. White blood cell (WBC) levels decreased starting on day 1, with the nadir occurring on days 14 – 15 for all dose groups before recovering back to pre-irradiation levels by day 30. Red blood cells (RBC), hemoglobin (HGB), and hematocrit (HCT) values followed a similar, but slower, pattern, where levels decreased after radiation exposure and increased back to baseline levels by the end of the study; however, recovery was delayed compared to WBC levels. Notably, the 7.2 Gy exposure group displayed a marginally worse recovery in RBC, HGB, and HCT values when compared to the other two dose groups.

Platelet levels declined sharply starting on day 6 in all three dose groups, with thrombocytopenia observed between days 12 – 17. Severe thrombocytopenia was noted in the 6.5 Gy exposure group on day 14 and in the 7.2 Gy exposure group between days 12 – 15. Platelet counts returned to pre-irradiation levels in all dose groups. Neutrophil counts also decreased sharply after irradiation, starting on day 2. Neutropenia was observed in the 7.2 Gy exposure group between days 5 – 19, and in the 5.8 and 6.5 Gy exposure groups between days 6 – 16. The 7.2 Gy exposure group exhibited the longest duration of severe neutropenia, lasting from days 10 – 16. The 6.5 Gy exposure group showed severe neutropenia from days 10 – 14, while the 5.8 Gy exposure group was severely neutropenic only on day 14. All dose groups showed recovery by day 60, but it is important to note that their neutrophil levels remained slightly below pre-irradiation levels at the end of the study.

Lymphocyte values showed a sharp decrease immediately following irradiation on day 1. A nadir was reached by day 16 in all dose groups before gradually returning back to near-baseline levels. Reticulocyte levels also declined starting on day 1 and recovery was observed around days 22 – 24 in all three dose groups. By days 28 – 30, reticulocyte values exceeded pre-irradiation levels before gradually declining back to baseline levels by day 60. Monocytes, eosinophils, and basophils did not follow any particular pattern for the three dose groups following irradiation.

### Effects of potentially lethal radiation exposures on serum biochemistry over the experimental time course

Eight parameters of blood chemistry were analyzed at various time points pre-and post-irradiation, which are shown in Supplementary Fig. 1. Significant differences between the radiation dose groups were found in most of the parameters at a few isolated time points. The 7.2 Gy exposure group exhibited the most variation across several parameters when compared to the two other dose groups.

Glucose levels were relatively stable in all three dose groups through day 38, with only a slight decrease after irradiation. However, by the end of the study, the 7.2 Gy exposure group showed decreased glucose levels, while the 5.8 and 6.5 Gy exposure groups returned to baseline levels. Albumin and total protein levels were similar across the three dose groups, remaining stable throughout the study, except in the 7.2 Gy exposure group, which exhibited slightly lower values when compared to the other two dose groups. Similarly, the 7.2 Gy exposure group demonstrated variation in gamma-glutamyl transferase (GGT) levels from the other two dose groups. GGT levels in the 7.2 Gy exposure group showed a sharp increase between days 38 – 60, while the 5.8 Gy and 6.5 Gy exposure groups had stable values throughout the study. Alanine aminotransferase (ALT), aspartate aminotransferase (AST), alkaline phosphatase (ALKP), and total bilirubin did not show consistent patterns across the three dose groups following irradiation.

### Effects of potentially lethal radiation exposures on serum cytokines over the experimental time course

Seven parameters were analyzed at various time points pre- and post-irradiation, which are shown in Supplementary Fig. 2. Significant differences were observed in all parameters, with the majority between the 7.2 Gy exposure group and the other two dose groups. Overall, significant differences were observed in cytokine responses following 5.8, 6.5, and 7.2 Gy irradiation. The cytokines induced within the narrow window of three days after radiation exposure can serve as exposure biomarkers. The selected cytokines were based on anecdotal experience over the years. and these cytokines have been shown to be useful biomarkers.

Interleukin-8 (IL-8) levels were very similar between the 5.8 and 6.5 Gy exposure groups, but the 7.2 Gy exposure group showed significantly higher values across all timepoints. This pattern was also seen in IL-6 and IL-1β. Interestingly, for granulocyte colony-stimulating factor (G-CSF), granulocyte–macrophage colony-stimulating factor (GM-CSF), and tumor necrosis factor-α (TNF-α), the 5.8 Gy exposure group had significantly higher values than the other two dose groups at all timepoints. IL-10 did not show any particular pattern for the three dose groups following irradiation.

## Materials and methods

### Experimental design

A total of thirty-nine rhesus NHPs divided into three groups were used in this study: 15 NHPs received 5.8 Gy (10 males, 5 females), 16 NHPs received 6.5 Gy (6 males, 10 females), and 8 NHPs received 7.2 Gy (5 males, 3 females).

### Animals

Naïve rhesus macaques (*Macaca mulatta*, Chinese sub strain) were obtained from Primate Products, Inc. (Miami, FL) and quarantined for 7 weeks prior to the initiation of the study. These clinically healthy male and female rhesus macaques were between 2.8 – 6.3 years of age, equivalent to about 11 – 19 years of age in humans^26^, weighing 3.6 to 8.9 kg. The experiments with three different doses were conducted sequentially, not concurrently. As such, the 7.2 Gy dose group had a fewer number of animals for the study. This was due to supply chain issues, resulting in limited availability of rhesus macaques at the time of this study. These animals were procured at different times based on the body weight and age range stated in the IACUC protocol. Thus, there was no randomization of animals among the three groups. All NHPs were individually housed in cages in environmentally controlled rooms maintained at 22 °C ± 2 ºC with 30 – 70% relative humidity, 10 – 15 air change cycles per h, and 12 h light:12 h dark cycles. Animals were fed primate diets twice per day and received drinking water ad libitum. All of the animals were able to see, hear, and/or touch the conspecifics throughout the cages. NHPs were stratified by sex and body-weight increases during the quarantine period, and were then assigned to different groups. The study animals were housed in a vivarium fully accredited by the AAALAC International^27^.

### Total-body irradiation

**Fig. 5:**
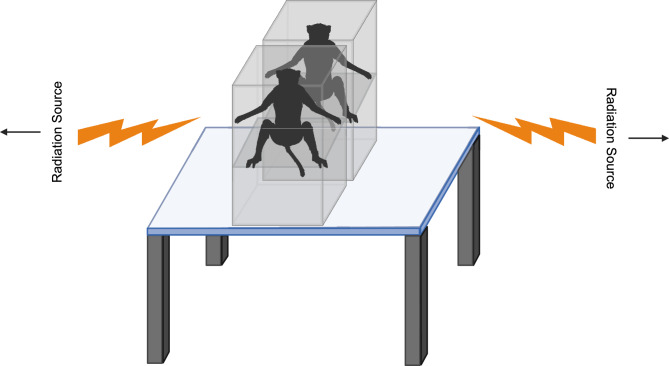
Diagram of the bilateral simultaneous irradiation procedure. Two NHPs were placed in custom-made restraint boxes on the irradiation platform facing back-to-back. The radiation sources were located on both sides of the NHPs. Animals were exposed to ^60^Co radiation bilaterally from both sources simultaneously for a targeted dose of 5.8 (n = 15), 6.5 (n = 16), or 7.2 (n = 8) Gy at a dose rate of 0.6 Gy/min.

The AFRRI high-level cobalt facility (HLCF) is a unique resource with two panoramic radiation sources which can be used either for bilateral simultaneous or unilateral sequential exposure approximating the procedures that can be replicated using a LINAC or point ^60^Co source where a rotation is required midway through exposures to obtain total-body irradiation. A maximum of eight NHPs were irradiated per day. Food was withheld for approximately 12 – 18 h before irradiation to minimize radiation-induced emesis. Approximately 30 – 40 min before radiation exposure, animals were administered 10 – 15 mg/kg of ketamine intramuscularly (*im*) for sedation. Two NHPs were placed on the irradiation platform facing away from each other and exposed with midline doses of 5.8, 6.5, or 7.2 Gy (bilateral simultaneous) at a dose rate of 0.6 Gy/min (Fig. 5). The irradiation field is uniformed within 3%. The targeted dose was delivered to the abdominal core of the animals. The difference between the two positions of the abdominal cores was less than 0.5%, which is within the measurement’s uncertainty of 1.5%. Dose rate measurements were primarily based on the alanine/EPR (electron paramagnetic resonance) system^28^.

### Medical management/symptomatic care

The type of supportive care provided was based on CBC analysis and cage-side observations (Supplementary Table 1)^29,30^. The primary antibiotic used was Baytril administered at a dose of 5 mg/kg *im* or subcutaneously (*sc)* twice daily, or 10 mg/kg administered *im* or intravenously (*iv)* once daily (QD). If body temperature was > 39.4 °C (in addition to neutrophil count < 500 cells/µL), ceftiofur was administered at 5 mg/kg *sc* for 2 days or 20 mg/kg for 7 days. Alternatively, gentamicin sulfate (5 mg/kg, *im* or *iv*, QD) was administered in combination with Baytril. If high fever persisted, ceftriaxone (50 mg/kg, *im*, every 24 h) was administered after discontinuing gentamicin sulfate or if microbial resistance to Baytril or gentamicin was demonstrated. Additional supportive care included rehydration fluids, antipyretics, alternate antibiotics, antidiarrheal agents, antiemetics, analgesics, treatment for mucosal ulcers, and nutritional support^29^.

### Blood sample collection

Blood was collected from the saphenous vein of the lower leg, distal to the knee. For venipuncture, a tourniquet was applied to the hind leg below the knee, and the site was cleaned using a 70% isopropyl alcohol wipe and dried with sterile gauze. For serum separation for blood chemistry and cytokine analysis, the blood samples were transferred to Capiject serum separator tubes, allowed to clot for 30 min to facilitate serum separation from cellular components, and then centrifuged at 1,000 g for 10 min. Samples for CBC, chemistry, and cytokines were collected as discussed earlier^31^. The serum samples for cytokine analysis were stored at -70 ºC until use. Blood samples for CBC were collected in EDTA tubes. Samples for CBC and blood chemistry were analyzed within 1 – 4 h after collection.

### CBC, blood biochemistry, and cytokine analysis

CBC analysis was performed using a Bayer Advia-120 hematology analyzer (Siemens Medical Solutions, Malvern, PA), while serum blood chemistry was analyzed using a Vitros 350 automatic biochemistry analyzer (Ortho Clinical Diagnostics, Markham, ON, Canada)^30^. Additionally, a Luminex 200 analyzer (Luminex Corp, Austin, TX) was used to measure chemokines, cytokines, and growth factors in the serum samples using custom-made kits (Bio-Rad Laboratories, Hercules, CA)^29^. Standard curves were prepared by serial dilution and ran as duplicate samples. Cytokine concentration was measured by fluorescence intensity, and its quantification was performed by using the Bio-Plex Manager software, version 6.1 (Bio-Rad Inc.).

### Euthanasia criteria

Euthanasia was conducted following the most recently approved versions of the IACUC protocol, the Guide, and the American Veterinary Medical Association (AVMA) guidelines^27,32^. When an animal reached a state of moribundity, the animal was euthanized. Moribundity was used as a surrogate for mortality, and animals were euthanized in order to minimize pain and distress^30^. The following parameters were used as guidelines for moribundity: significant weight loss (10%) from baseline; inappetence (complete anorexia for 2 days and deteriorating conditions); minimal or absence of response to stimuli, severe anemia (< 13% hematocrit due to acute blood loss or < 40 g/dL hemoglobin) and core body temperature below 36 °C following a period of febrile neutropenia (such as > 39 °C and < 500 neutrophils/µl); weakened/inability to obtain feed or water; severe thrombocytopenia (< 10,000 platelets/µl) or other signs of severe organ dysfunction with poor prognosis as determined by the veterinarian such as dyspnea or severe cyanosis; sustained vomiting or diarrhea, obstruction, intussusception and peritonitis; renal failure as determined by clinical chemistry and urinalysis; sustained CNS depression, seizures, or paralysis of one or more extremities; non-healing wounds, repeated self-trauma, and severe skin infections; and severe organ system dysfunction with poor prognosis. Any single parameter from the above-listed guidelines did not lead to euthanasia of any animal. Moribundity status of the animal was determined by a joint effort between the institutional veterinarian, principal investigator, research staff, veterinary technicians, and husbandry staff based on the combination of criteria described above. The moribund animals were given pentobarbital sodium *iv* (Virbac AH Inc., Fort Worth, TX) using either saphenous or cephalic veins, needle size 20 – 25 gauge (100 mg/kg, 1 – 5 ml). Prior to pentobarbital sodium administration, animals were sedated using ketamine hydrochloride injection (Mylan Institutional LLC, Rockford, IL) (5 – 15 mg/kg, *im*). Intra-cardiac administration was performed if unable to administer pentobarbital sodium through peripheral veins. The animals were deeply anesthetized by Isoflurane (Baxter Healthcare Corporation, Deerfield, IL) (1 – 5%) with oxygen at 1 – 4 L per minute via mask before administering the intra-cardiac injection. The animals were euthanized only under the guidance of a staff veterinarian or a trained technician in consultation with the veterinarian. After pentobarbital sodium administration, the animals were examined by assessing the heart auscultation and pulse to confirm death.

### Statistical analysis

Statistical software IBM SPSS Statistics version 29.0.2.0 was used for all statistical analyses. A minimum of three animals per radiation dose group was required to determine significance at any given time point. All error bars represent standard errors of the mean (SEM). One-way analysis of variance (ANOVA) tests with the Tukey–Kramer post-hoc test was used to identify significant differences in CBC, blood chemistry, cytokine responses, and vital signs between the three groups. Chi-square tests were performed on the survival data to compare survival rates of each exposure group, as well as to compare males and females in each exposure group. A p-value of < 0.05 was considered statistically significant for all tests performed.

### ARRIVE compliance statement

The authors confirm the current study was conducted in compliance with ARRIVE guidelines.

## Discussion

Currently, NHPs, in particular rhesus macaques, play a pivotal role in evaluating the efficacy of MCMs as based by their countering effects on overall morbidity and mortality in animals with ARS induced by a range of potentially lethal radiation exposures^17^. This study clearly shows graded, radiation dose-dependent responses of adult rhesus macaques following a series of potentially lethal exposures (i.e., 5.8, 6.5, and 7.2 Gy) that approximate or border a mid-lethality range (i.e., LD_50/60_). These findings are not unexpected, but consistent with the findings from other comparable studies and, thus, provide additional functional support for this NHP model^17^.

There are several other animal models in addition to NHPs such as ferrets, swine (specifically minipig), canines, rabbits, and rats that are used to study MCM efficacy and radiation pathophysiology, although some are more suitable for particular endpoints. For example, ferrets are used for studying emesis, while minipigs are ideal for investigating cutaneous injuries^33–36^. Because of the ethical reasons of testing the efficacy of a drug in countering the effects of ionizing radiation in normal, healthy individuals, the FDA developed the Animal Rule that allows new agents to be tested for efficacy using well-understood animal models^25,37–39^. The recommendation is that not only should large animal models be used during advanced preclinical testing, but both sexes should be included. The limited availability, high purchase cost, and maintenance of a required number of large animals of both sexes significantly increase the cost of completing these studies, accentuating the financial burden and logistical challenges associated with conducting such studies.

Despite NHPs’ extensive use in studies of the pathophysiology of acute radiation exposures, prior studies have reported considerable heterogeneity in radiation dose–response relationships. This variability can result from differences in radiation sources, the level of clinical support provided, radiation qualities, and other environment factors across studies^21,40^. Factors such as animal sex, medical management, and the use of blood transfusions can also influence the dose–response relationship, meaning that variations in these factors could also lead to differences in the observed efficacy and toxicity of MCMs as well. Consequently, data on radiation-induced responses that stem from various radiation sources and varying radiation qualities, or the levels/types of medical support, are often limited. Furthermore, comparing such data across laboratories is further complicated by inconsistencies in experimental design and the reporting of study methodologies^21,23,41,42^.

Different lethality responses of acutely irradiated NHPs have been reported previously when different levels of supportive care (e.g., the use of blood transfusions) have been provided post-exposure. Further, these studies were not generally conducted under exactly the same experimental conditions^21^. In brief, the exact benefit of supportive care, including the use of blood products, needs to be established. In this regard, we have reported previously lethality responses using rhesus NHPs exposed to total-body radiation (^60^Co gamma rays) and subsequently cared for with a full regimen of supportive treatments, including blood transfusions. From these lethality responses, a set of ‘lethal dose’ (LD) values were established: these determined values were 5.71 Gy for LD_30/60_ , 6.78 Gy for LD_50/60_, and 7.84 Gy for LD_70/60_^29^ . These doses represent the calculated lethality value for 30%, 50%, and 70% mortality, respectively, within 60 days. By comparison, in the current study where a component of supportive care, i.e., blood transfusions, was omitted, a LD_50/60_ value of 7.0 Gy was approximated. This difference is small compared with the value reported in literature. Such variability may be because these studies, either ours or referred in the literature, have not been conducted concurrently under exactly the same experimental conditions with the same source of animals.

Both the previous and the current study used similar experimental conditions, except for different levels of supportive care provided. Although these two studies were not concurrently conducted, the demonstrated lethality responses of the animals were similar and the mix of the two sexes within specific dosing groups did not seem to seriously impact survival outcomes.

Concerning the issue of sex-based response differences, the majority of available data sets come from studies using male-only NHPs for total-body irradiation. This reliance on a single sex results in an incomplete understanding of the biological processes affected by total-body irradiation, as sex-specific differences may not be captured. At least two reports that have reviewed numerous studies have suggested that female NHPs are more sensitive to radiation exposure, showing higher mortality when compared to males and differences in hematological profiles, including distinct CBC nadirs^31,42^. A similar result of higher mortality in females was observed in this study, however, unfortunately, this study cannot directly confirm or deny these sex-dependent findings due to the limited number of animals of both sexes that had been assigned to individual radiation dose groups. All of these findings, both earlier and current ones, are particularly important for the identification and treatment of ‘most-in need’ radiation-exposed ARS victims during mass casualty radiological/nuclear events, especially in situations where critical medical resources may be limited. In the current study, we have included both male and female animals and these responses noted are with total-body radiation exposures and not partial-body exposures. In future studies, it will be important to investigate whether there are persistent response differences between the sexes under different exposure scenarios (e.g., under partial-body irradiation). While our current study included NHPs of both sexes, sample size was not sufficient to fully assess sex-specific differences.

As there are numerous other critically important variables (e.g., such as radiation source/quality, exposure geometry, supportive care regimen, etc.) that can influence response outcomes, the requirement to include both sexes into a single given experiment may represent an experimental obstacle most difficult to overcome and somewhat unrealistic when one considers the current constraints on NHP-based biomedical research. Regardless, the ‘good news’ is that progress indeed has been made in attempt to define acute radiation associated injuries within NHPs and to effectively counter those potential lethal injuries with various MCMs. Previous studies with rhesus macaques exposed to uniform total-body radiation doses with a ^60^Co gamma source in various institutions have shown relatively consistent dose–response relationships^8,21,42–45^, although the role of supportive care, particularly the use of blood products, remains a debated issue from a feasibility perspective^42^. In the study reported here, NHPs were exposed to radiation in a HLCF model with bilateral simultaneous exposure. Again, as we indicated, there are some limitations in our study. For example, the number of male and female animals across the three radiation doses were not equal. This was due to the limited availability of such animals at various times and this was especially true for females. Consequently, we had only eight animals in the 7.2 Gy group compared to 15 and 16 animals for the other two lower exposure groups, i.e., 5.8 and 6.5 Gy, respectively. Independent of these issues, we believe that the current study has foundational value in our attempt to establish baseline exposure requirements for both sexes under post-exposure conditions that exclude extensive medical management. In the future, studies may be conducted with an equal number of males and females in groups of each radiation dose. Since such studies will be specifically useful for servicemembers on the frontline battle field, it may be better to use limited and common antibiotics as Baytril.

Well-defined large animal models are critical for the development and regulatory approval of effective MCMs for various sub-syndromes of ARS and the delayed effects of acute radiation exposure. The NHP model is especially important for securing regulatory approval for MCMs from the US FDA for human use under the Animal Rule^25^. This study aimed to further characterize the rhesus macaque model using both male and female animals in the AFRRI HLCF to investigate radiation injury and support the development and regulatory approval of MCMs. As expected, the data demonstrated radiation dose-dependent injury in the animals. Taken together, the results of this study will provide valuable insights for conducting ^60^Co gamma-radiation exposure tests in NHPs to evaluate radiation MCMs, particularly in scenarios where blood products are not used.

## Supplementary Information

Supplementary Information.

## Data Availability

Data is provided within the manuscript or supplementary information files.
