# Supplementary material for: Characterization of rhesus macaque model for cobalt-60 gamma-radiation source without use of blood product
Source: Sci Rep. 2025 Aug 26;15:31378. doi: 10.1038/s41598-025-17099-7 (PMC12381090; doi:10.1038/s41598-025-17099-7)
Supplement: Supplementary file 1 — Supplementary Information. [file 41598_2025_17099_MOESM1_ESM.pdf]

Supplementary Table 1. Details of medical management/symptomatic palliative care

Drug class	Allowed medication or supportive care agents	Indication and/or criterion for administration
Parenteral fluids	Lactated Ringer's Solution (LRS) or LRS with 5% Dextrose; each at 5 ± 2.5 mL/kg body weight via *sc* route; up to twice daily depending on extent of dehydration. Fluid administration rates and volumes change at the discretion of the veterinarian<br><br>Bottles containing diluted fruit juice or oral rehydration solution (Prang™, Bio-Serv) provided 10 – 20 d post-irradiation and refilled as needed.	Dehydration: Mild to moderate dehydration signs and symptoms include subtle loss of skin elasticity (observed by skin turgor while in restraint chair), decreased urine output, and increased thirst<br><br>Severe dehydration signs and symptoms: rapid breathing, lethargy, severe loss of skin elasticity, sunken eyes, dry buccal mucous membranes, extremities cool to the touch, decreased body temperature. Treatment options at discretion of veterinarian.
Topical antiseptic	Chlorohexidine oral spray applied once daily while in restraint chair for blood collection.	For treatment of mouth ulcers as observed while in the restraint chair
Anti-emetics	Ondansetron (Hospira Inc., Lake Forest, IL) 1 – 2 mg/kg, *im,* 25 – 90 min prior to irradiation and 30 – 45 minutes following irradiation	Administered pre-and post-irradiation to suppress emesis and nausea.

Nutritional Support	To entice the consumption of food post-irradiation, the primates were provided with an assortment of food that heightened their interest. Such items included biscuits soaked in Ensure, yogurt, dried fruit, popcorn, cookies, crackers, marshmallows, cereals, pasta, frozen Gatorade cubes, mixed nuts, seeds, etc.<br><br>Fresh items provided included: apples, bananas, peppers, carrots, celery, oranges and sweet potato. This brief listing is not meant to exclude any food product that was in the regular diet for the animals.	Animals received daily enrichment and additional treats following blood collection. During d 10 – 20 post-irradiation, if biscuits (normal ration) were not consumed, biscuits soaked in Ensure were provided at the next scheduled feeding.
Analgesics	Buprenorphine HCL (Hospira Inc., Lake Forest, IL) 0.01 – 0.02 mg/kg *im,* BID<br><br>Carprofen (Rimadyl, Zoetis LLC, Parsippany, NJ) 15 mg/tab BID, *po* or 1 – 5 mg/kg BID, *sc*<br><br>Meloxicam SR (Wedgewood Pharmacy, Swedesboro, NJ) 0.6 mg/kg, *sc*, every three days	Body temperature >39.4 °C (fever) or visual signs of pain (hunched appearance, limited/slow movement while in the cage) as determined by the veterinarian.
Antibiotics	Enrofloxacin (Baytril, Bayer HealthCare LLC, Shawnee Mission, KS) 5 +/ 0.25 mg/kg *im* or *sc*, twice a day (BID); or 10 +/- mg/kg *im* or *sc* once daily (QD). Other antibiotics provided at the discretion of the veterinarian<br><br>Ceftiofur (Excede, Zoetis LLC, Parsippany, NJ) 5 mg/kg *sc* for 2 days or 20 mg/kg for 7 days<br><br>Gentamicin sulfate (GentaMax®, Clipper Distributing Co., LLC, Phoenix Pharmaceutical, Inc., St. Joseph, MO) 5 mg/kg, *im* or *iv*, QD<br><br>Ceftriaxone (Pfizer Inc., New York, NY) 50 mg/kg, *im*, every 24 h	ANC (absolute neutrophil count): Antibiotic regimen was initiated if the ANC was <500/µL and was continued until ANC reached >500/µL.<br><br>Body temperature >39.4 °C

Subcutaneous administration: *sc*; intramuscular administration: *im*; oral administration: *po*.

Supplementary Figure 1

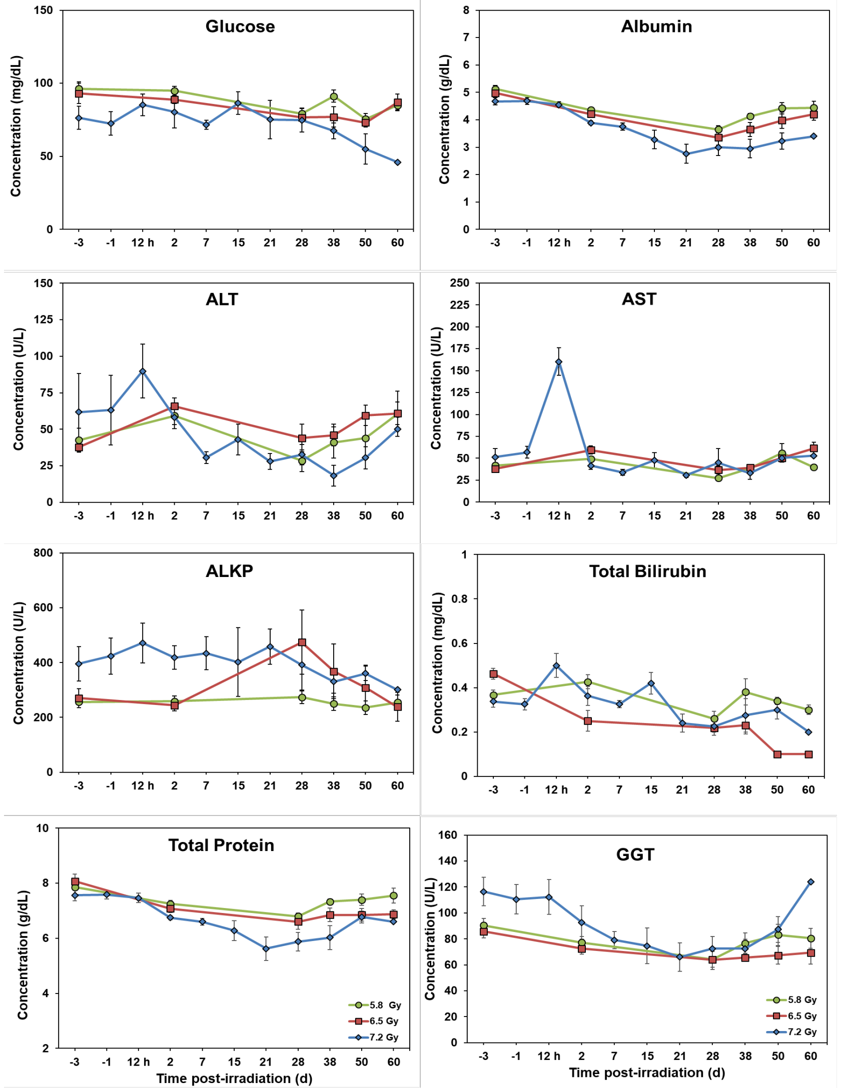

Parameter	Comparison	Days post-irradiation and significance
Albumin	5.8 vs 7.2 Gy	2, 38, 50*
AST	6.5 vs 7.2 Gy	2*
ALKP	5.8 vs 7.2 Gy<br>6.5 vs 7.2 Gy	2***<br>2***
Total bilirubin	5.8 vs 6.5 Gy<br>6.5 vs 7.2 Gy	-3*, 2**, 50***<br>-3**, 50***
Total protein	5.8 vs 7.2 Gy	38**
GGT	5.8 vs 7.2 Gy<br>6.5 vs 7.2 Gy	-3*<br>-3*

Supplementary Figure 1. Effects of 5.8 (n=15), 6.5 (n=16), or 7.2 (n=8) Gy ^{60}Co total-body γ-radiation on glucose, albumin, alanine aminotransferase (ALT), aspartate aminotransferase (AST), alkaline phosphatase (ALKP), total bilirubin, total protein, and gamma-glutamyl transferase (GGT). Data for each time point are presented as the mean for each group. Statistical significance is presented in the table below the graphs and are denoted by asterisks where *$p<0.05$, **$p<0.01$, and ***$p<0.001$. Error bars represent SEM.

Supplementary Figure 2

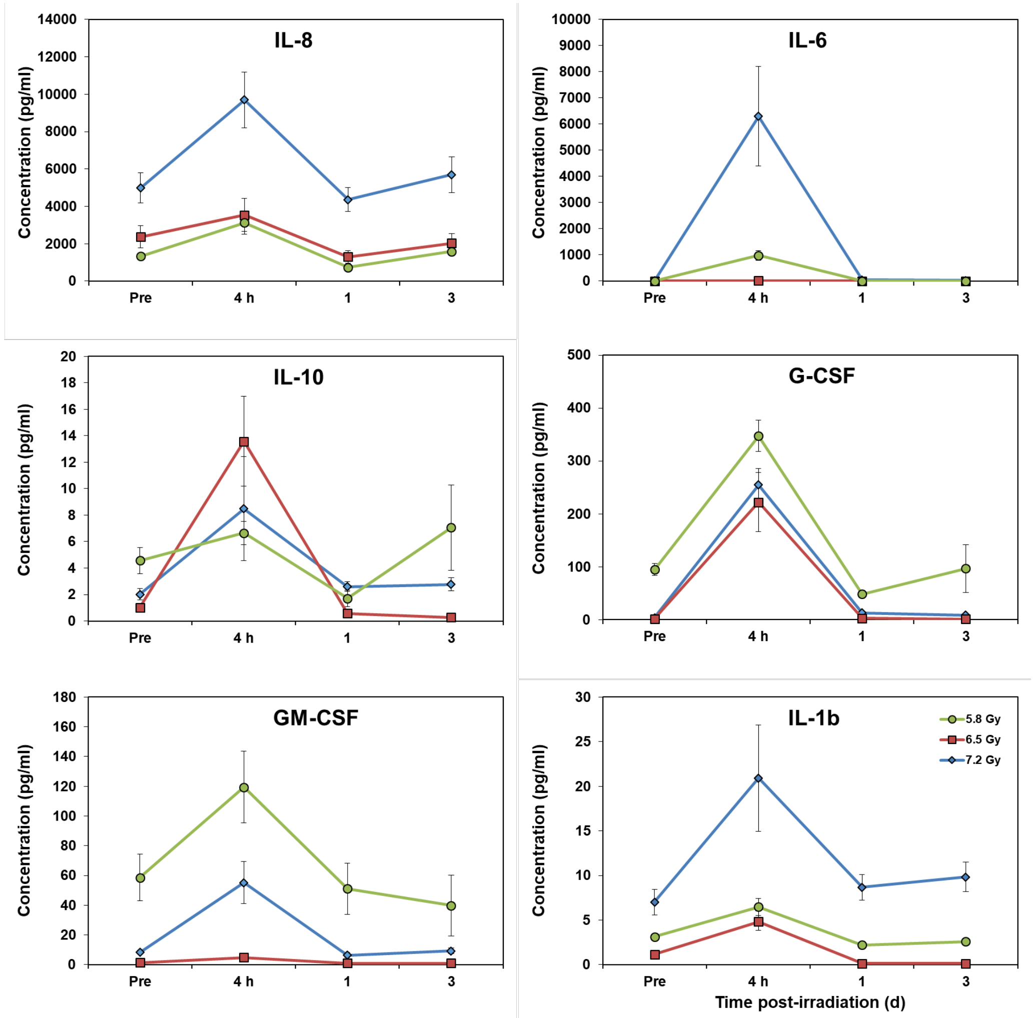

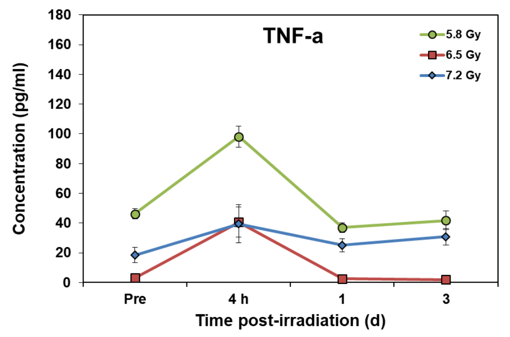

Parameter	Comparison	Days post-irradiation and significance
IL-8	5.8 vs 7.2 Gy	Pre***, 4 h***, 1***, 3***
	6.5 vs 7.2 Gy	Pre***, 4 h***, 1***, 3***
IL-6	5.8 vs 7.2 Gy	Pre***, 4 h***, 1***, 3***
	6.5 vs 7.2 Gy	Pre***, 4 h***, 1***, 3***
IL-10	5.8 vs 6.5 Gy	Pre**
	6.5 vs 7.2 Gy	1*
G-CSF	5.8 vs 6.5 Gy	Pre***, 4 h*, 1***
	5.8 vs 7.2 Gy	Pre***, 1***
GM-CSF	5.8 vs 6.5 Gy	Pre***, 4 h***, 1**
	5.8 vs 7.2 Gy	Pre*, 1*
IL-1b	5.8 vs 6.5 Gy	1**, 3**
	5.8 vs 7.2 Gy	Pre*, 4 h**, 1***, 3***
	6.5 vs 7.2 Gy	Pre***, 4 h**, 1***, 3***
TNF-a	5.8 vs 6.5 Gy	Pre***, 1***, 3***
	5.8 vs 7.2 Gy	Pre***, 1*
	6.5 vs 7.2 Gy	Pre**, 1***, 3***

Supplementary Figure 2. Effects of 5.8 (n=15), 6.5 (n=16), or 7.2 (n=8) Gy ^{60}Co total-body γ-radiation on interleukin-8 (IL-8), IL-6, IL-10, granulocyte colony-stimulating factor (G-CSF), granulocyte-macrophage colony-stimulating factor (GM-CSF), IL-1β, and tumor necrosis factor-α (TNF-α). Data for each time point are presented as the mean for each group. Statistical significance is presented in the table below the graphs and are denoted by asterisks where *p<0.05, **p<0.01, and ***p<0.001. Error bars represent SEM.
